# Developing a Fluorescent Inducible System for Free Fucose Quantification in *Escherichia coli*

**DOI:** 10.3390/bios13030388

**Published:** 2023-03-15

**Authors:** Samantha Nuñez, Maria Barra, Daniel Garrido

**Affiliations:** Department of Chemical and Bioprocess Engineering, School of Engineering, Pontificia Universidad Católica de Chile, Vicuña Mackenna, 4860, Santiago 8331150, Chile

**Keywords:** biosensor, *Escherichia coli*, fucose, GFP, gene regulation

## Abstract

L-Fucose is a monosaccharide abundant in mammalian glycoconjugates. In humans, fucose can be found in human milk oligosaccharides (HMOs), mucins, and glycoproteins in the intestinal epithelium. The bacterial consumption of fucose and fucosylated HMOs is critical in the gut microbiome assembly of infants, dominated by *Bifidobacterium*. Fucose metabolism is important for the production of short-chain fatty acids and is involved in cross-feeding microbial interactions. Methods for assessing fucose concentrations in complex media are lacking. Here we designed and developed a molecular quantification method of free fucose using fluorescent *Escherichia coli*. For this, low- and high-copy plasmids were evaluated with and without the transcription factor *fucR* and its respective fucose-inducible promoter controlling the reporter gene sfGFP. *E. coli* BL21 transformed with a high copy plasmid containing *pFuc* and *fucR* displayed a high resolution across increasing fucose concentrations and high fluorescence/OD values after 18 h. The molecular circuit was specific against other monosaccharides and showed a linear response in the 0–45 mM range. Adjusting data to the Hill equation suggested non-cooperative, simple regulation of FucR to its promoter. Finally, the biosensor was tested on different concentrations of free fucose and the supernatant of *Bifidobacterium bifidum* JCM 1254 supplemented with 2-fucosyl lactose, indicating the applicability of the method in detecting free fucose. In conclusion, a bacterial biosensor of fucose was validated with good sensitivity and precision. A biological method for quantifying fucose could be useful for nutraceutical and microbiological applications, as well as molecular diagnostics.

## 1. Introduction

L-Fucose is an important monosaccharide that exerts functional roles in multiple biological processes [1]. L-Fucose is a deoxy hexose sugar characterized by missing a hydroxyl group at C-6 [1]. It is commonly present in mammalian mucins, human milk oligosaccharides (HMOs), and glycoconjugates of the intestinal epithelium [2,3], such as glycolipids, N-glycans, and O-glycans. In these glycoconjugates, fucose is usually found at terminal positions in α- linkages (such as α1-2, α1-3, α1-4, and α1-6; [4]). The main enzyme responsible for these fucosylations is α-1,2-fucosyltransferase (Fut2), which is expressed in epithelial cells and links fucose to the terminal β-D-galactose of mucosal glycans [5]. 

Fucose is abundant in the gastrointestinal tract (GT) and influences the complex microbial ecosystem that inhabits there. Several microorganisms are equipped with α-fucosidases targeting all existing fucose linkages [6,7,8]. Therefore, gut microbes can release fucose from dietary glycans, which is used as a microbial carbon source [9]. In addition, fucose may promote the growth of beneficial bacteria in the gut, such as *Bifidobacterium* and *Bacteroides* [10]. Fucose is finally a common exchange molecule involved in multiple microbial cross-feeding interactions [11,12,13].

In addition to serving as an energy source for some microbes, fucose is involved in diverse metabolic pathways, including the regulation of quorum sensing and suppression of virulence genes in pathogens [14,15]. Detecting low levels of free fucose in biological samples could be a valuable indicator of infection or inflammation [16]. Some pathogens can use fucose as a signaling molecule regulating pathogenesis [17]. Fucose and fucose-containing oligosaccharides usually act as a decoy, preventing the binding of some viral and bacterial pathogens [18]. High levels of fucose in urine have been associated with cirrhosis and certain types of cancer [19,20]. Finally, loss of function mutations of fucosyl-transferase Fut2 have been associated with Crohn’s disease [21]. 

Free fucose is usually quantified by HPLC [22,23,24,25] and enzymatic assays with L-fucose dehydrogenase [19,26]. Recently, lectin-based microfluidic detection assays have been developed [27,28], as well as fluorescence-based assays with probes and electrochemical sensors [20,29]. Shin et al. [30] developed a molecular biosensor for quantifying 2-fucosyllactose (2FL) in breast milk samples. Their circuit contained a constitutive α-fucosidase expressed in an *E. coli* strain mutant for lactose consumption. Therefore, the detection of 2FL and emission of fluorescence were coupled to cell growth and 2FL degradation [30].

Bacterial biosensors are genetically modified organisms that detect an input, usually a substance or the changes in the concentrations of a specific molecule, which are sensed and internally translated into a genetic output that emits a quantifiable signal [31,32]. Bacterial biosensors are usually constructed of transcription factors and their corresponding promoters. The most common outputs are fluorescent proteins such as Green Fluorescent Protein (GFP). An excellent candidate to develop a bacterial biosensor is *Escherichia coli*, a model widely used in biotechnological research and development since its genome and metabolic pathways are fully known [30,32,33]

*Escherichia coli* K12 can use multiple sugars as a carbon source for its growth, including fucose [34,35]. Fucose can induce the expression of genes allowing its transport and metabolism, a genetic system known as the fucose regulon. It consists of six genes: L-fucose permease (*fucP*), L-fucose isomerase (*fucI*), L-fuculose kinase (*fucK*), L-fuculose phosphate aldolase (*fucA*), L-1,2-propanediol oxidoreductase (*fucO*) and the transcription regulator of the regulon (*fucR*) [36,37]. These genes are clustered into three operons, fucPIK, fucA (which is transcribed in a clockwise direction), and fucO (which is transcribed counterclockwise). FucR is an activator [37], which is induced by fuculose 1-phosphate, an intermediate molecule from fucose metabolism. FucR also shows positive autoregulation [38]. 

In this study, we used the *fuc* molecular system for developing a method of quantifying free L-fucose, using FucR and the fuc promoter triggering the induction of sfGFP in *E. coli.* We first compared the detection of fucose in high- and low-copy plasmids with or without *fucR.* The best system was evaluated for specificity, and calibration curves were obtained at low (0–3 mM) and high concentrations (0–50 mM) with good resolution. The biosensor was successfully applied to quantify fucose in bacterial supernatants. This molecular biosensor could be further studied to quantify free fucose in complex biological samples with good resolution and specificity. 

## 2. Materials and Methods

*Mediums, reagents, and sugars.* Miller Luria-Bertani (LB) liquid and agar medium was obtained from Merck (Boston, MA, USA) and autoclaved at 121 °C for 15 min. Minimum medium MM9 was prepared with KH_2_PO_4_ (15% *w*/*v*), NaCl (2.5% *w*/*v*), Na_2_HPO_4_ (33.9% *w*/*v*), and NH_4_Cl (5% *w*/*v*). These reagents were obtained from Sigma Aldrich (St. Louis, MO, USA). The liquid medium ZMB was prepared according to Medina et al. [39]. Solid media contained 1.5% *w*/*v* agar. Carbohydrates used were L-fucose, 2-O-fucosyllactose, 3-O-fucosyllactose, and sialic acid (Neu5ac), which were kindly donated by Glycom (Hørshol, Denmark). Mannose, glucose, galactose, and lactose were obtained from Sigma Aldrich (St. Louis, MO, USA). Carbohydrate solutions were prepared with Milli-Q water and then filtered with Millex-gv filters (0.22 μm).

*Plasmid construction.* The in-silico plasmid construction was carried out in the SnapGene program, obtaining all the constructs and the primers (Table 1). Fucose biosensors were created in a high copy plasmid backbone (pTAC_sfGFP ColE1) and a low copy plasmid backbone (pTAC_sfGFP SC101). The high copy plasmid contains an ampicillin resistance gene as a selection marker and superfolder GFP (sfGFP) as a reporter molecule [40], while the low copy plasmid contains a chloramphenicol resistance and sfGFP controlled by *pTac*. These plasmids were a kind donation from Dr. Tal Danino (Columbia University). The fucose-induced promoter (*pFuc*) was synthesized as a gBlock from Integrated DNA Technologies, Inc. (IDT), including the PstI and EcoRI restriction sites at the 5′ and 3′ ends, respectively. The sequence was obtained from the *E. coli* K12 MG1655 genome, specifically from the fucose *fucPIK* operon [38]. Both DNA fragments were digested with the PstI-HF and EcoRI-HF restriction enzymes for 1 h at 37 °C, gel purified, and ligated with T4 DNA ligase at room temperature for 1 h (New England Biolabs, Inc., Ipswich, MA, USA). The resulting plasmids were named pFUC_sfGFP_colE1 and pFUC_sfGFP_SC101.

*Cloning of FucR.* Later, the transcription factor gene (*fucR*) was obtained from the genome of *E. coli* K12 MG1655 by PCR with the primers 5′-tctcatACCGGTacgcccgcc-3′ and 5′-ctatCCCGGGtcaggctgttaccaaagaag-3′. These primers contain restriction sites for the enzymes *AgeI* and *XmaI*. PCR reactions were performed with Q5 high-fidelity polymerase (New England BioLabs, Ipswich, MA, USA) using manufacturer instructions. Exceptions were an annealing temperature of 70 °C and an extension time of 20 s, using 0.5 μM of the primers and 1 U of polymerase Q5. PCR products were recovered from a 1% agarose gel with the Zymoclean Gel DNA Recovery Zymo research kit (Irvine, CA, USA). The pFUC_sfGFP_colE1 plasmid containing the fuc promoter was digested with the same enzymes and amplified with the primers 5′-TGAcgctagaactagtggatcc-3′ and 5′-tcagACCGGtagaccgagatagggttgag-3. PCR products were recovered (FucR and the fucose-induced promoter in the high-copy plasmid (Table 1)). Digestions were carried out for 16 h at 37 °C with *AgeI-HF* and *XmaI* enzymes following manufacturer instructions (New England Biolabs; Ipswich, MA, USA). Digested plasmids and fragments were ligated with a T4 DNA ligase (New England Biolabs; Ipswich, MA, USA) at room temperature for 16 h. 

*Bacterial transformations.* All plasmids were transformed into chemically competent *E. coli* strains. Plasmids were stored in DH5α, and biosensors were produced in the BL21 strain. Two microliters of ligation mixture or Gibson Assembly Master Mix were added to 50 µL of cells and incubated on ice for 30 min. Heat shock was performed at 42 °C for 50 s, followed by 2 min on ice. One ml of SOC media was added, and the bacteria were incubated at 37 °C with shaking at 200 rpm for 1 h. The transformation volume was plated onto LB agar plates with the corresponding antibiotic. Single colonies were picked and cultured in LB media with the antibiotic for stock preparation and miniprep. Carbenicillin was used at 100 µg/mL, and chloramphenicol was prepared in ethanol at 25 µg/mL. Correct insertion of genes of interest was verified through plasmid sequencing at Macrogen Inc (Seoul, Republic of Korea).

*Fluorescence kinetics.* Four colonies were selected per plate and cultured in 2 mL of LB-antibiotic broth with 200-rpm agitation for 16 h at 37 °C. Filtered fucose (100 mM) was used to prepare 200 μL triplicate reactions with decreasing monosaccharide concentrations, inoculated at 1% *w*/*v* with fresh LB-antibiotic medium. All kinetics were performed on black with transparent bottom Nunc™ F96 MicroWell™ 96-well polystyrene plates (Thermo Scientific, Waltham, MA, USA)), in a Synergy H1 Biotek multi-plate reader (Winooski, VT, USA). Growth curves were monitored for 24 h with agitation, measuring OD_600_ and fluorescence every 30 min with excitation at 485 nm and emission at 510 nm. The Gen5 3.09 software was used for absorbance and fluorescence measurements and data analysis. 

*B. bifidum culture and fucose quantification. B. bifidum* JCM 1254 was inoculated in de Mann Rogose Sharp (MRS) broth supplemented with cysteine 0.05% for 48 h in an anaerobic jar at 37 °C with an anaerobic GasPak EZ patch (Becton Dickson, Franklin Lakes, NJ, USA). Cells were centrifuged at 12,000× *g* for 1 min after 48 h and resuspended in reduced mZMB broth [39] with no carbon source. *B. bifidum* was then cultured at 4% *w*/*v* in 5 mL of mZMB supplemented with 2FL (81 mM) or with 3FL (20.4 mM) for 40 h under anaerobic conditions as above. Supernatants were recovered at 0, 12, 16, 20, 24, and 40 h. All supernatants were filtered with Millex-gv 0.22 μm filters (Sigma Aldrich, St. Louis, MO, USA), and pH was adjusted to 7 using NaOH 1 M. Supernatants were later analyzed using thin layer chromatography (TLC) in parallel to biosensor detection. Standards of fucose, 2FL, 3FL, lactose, galactose (all at 1% *w*/*v*), and *B. bifidum* supernatants JCM1254 were used. TLC DC-Fertigfolien ALUGRAM Xtra Silica Gel 0.20 mm plates were used (Macherey-Nagel, Allentown, PA, USA), with 1 μL of each sample. A run solution was prepared with 50% *v*/*v* of n-butanol and 25% *v*/*v* of acetic acid in distilled water. Two ascents were performed to improve resolution. A staining solution was prepared with 0.5% *w*/*v* naphthol and 5% *v*/*v* sulfuric acid in ethanol.

*Statistical analysis.* All curves represent the average of triplicates, and the standard deviation is shown. Statistical analyses, including linear regressions, were performed in GraphPad Prism 9. To determine the sensitivity of the regulation and potential cooperativity, the Hill equation for an activator was fitted to fluorescence/OD values [41]. Hill equation parameters were minimized to experimental data using Solver in Excel. β is the maximum expression rate, K represents the dissociation constant, [S] is the substrate concentration and n is the Hill cooperativity coefficient [42].

(1) $$\frac{d\;\left(F/OD\right)}{d\;t}=\frac{\beta K^{n}}{K^{n}+{\left[s\right]}^{n}}$$

## 3. Results

### 3.1. Biosensor Properties and Functions

The three plasmids constructed in this study are shown in Table 1 and depicted in Supplementary Figure S1. The reporter gene sfGFP is controlled by *pFuc* and is a marker of selection that gives resistance to antibiotics. Low- and high-copy plasmids were evaluated. The transcription factor gene *fucR* was also cloned upstream of *pFuc*, with constitutive expression. The plasmids were used for chemical transformations of the BL21 and DH5α *E. coli* strains and tested with increasing fucose concentrations. 

**Figure 1:**
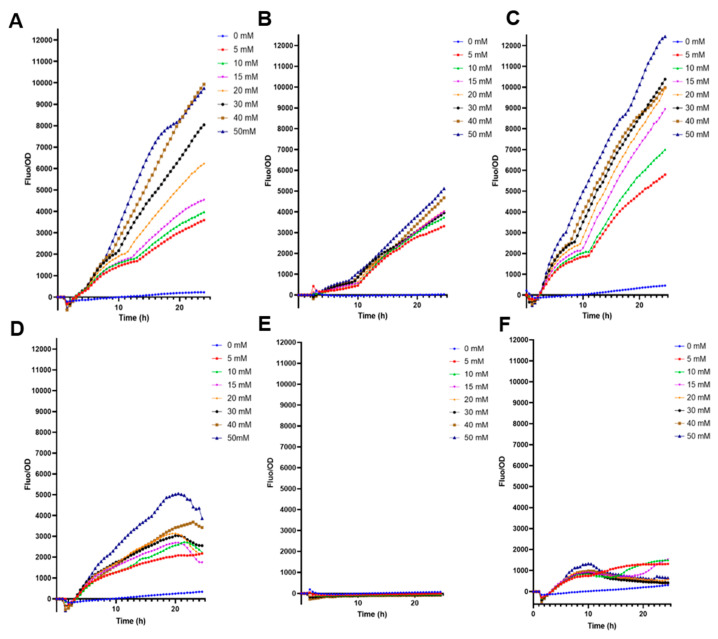
Fluorescence kinetics of different *E. coli* biosensors in response to increasing fucose concentrations. (**A**) *E. coli* BL21 containing pFuc_sfGFP_colE1; (**B**) *E. coli* BL21 containing pFuc_sfGFP_SC101; (**C**) *E. coli* BL21 containing pFuc_FucR1_colE1; (**D**) *E. coli* DH5α containing pFuc_sfGFP_colE1; (**E**) *E. coli* DH5α containing pFuc_sfGFP_SC101; (**F**) *E. coli* DH5α containing pFuc_FucR1_colE1. Kinetic curves were performed in triplicates.

*E. coli* BL21 transformed only with *pFuc* displayed good behavior with increased F/OD ratio to higher fucose concentrations (Figure 1A). This molecular system did not fluoresce with 0 mM fucose, suggesting a tight control (Figure 1A). Interestingly, the cloning of *fucR* and *pFuc* into the high copy plasmid increased fucose detection values by nearly 50%, with good resolution and increasing F/OD ratios in response to higher fucose concentrations in BL21 (Figure 1C). The low-copy plasmid biosensor (SC101) emitted smaller fluorescence values than high-copy plasmids (Figure 1B; *p* < 0.0001 at 50 mM fucose). The three plasmids transformed in *E. coli* DH5α generated F/OD curves that did not correlate well with fucose concentrations (Figure 1D–F).

### 3.2. Comparison of Biosensor Specificities

**Figure 2:**
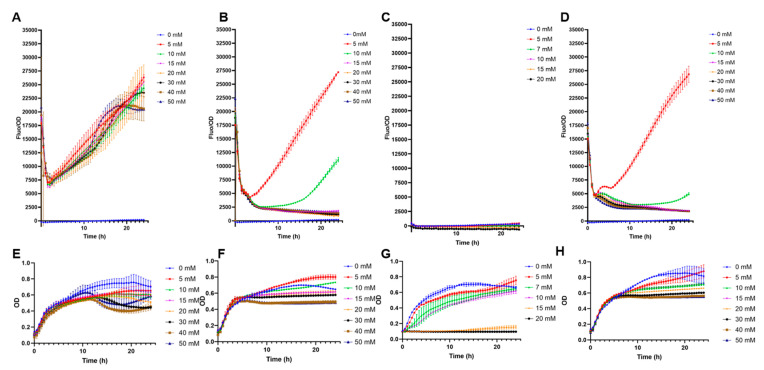
Specificity tests of *E. coli* BL21 containing pFuc_sfGFP_colE1. (**A**) F/OD rations in the presence of increasing concentrations of galactose; (**B**) glucose; (**C**) sialic acid; (**D**) mannose; (**E**) growth curves (OD values) of this strain in the presence of increasing concentrations of galactose; (**F**) glucose; (**G**) sialic acid; (**H**) mannose. Kinetics and growth curves were performed in triplicates and are presented as average ± SD.

High-copy biosensors with *pFuc* and pFuc+FucR in BL21 were preliminary evaluated for non-specific cross-detection of other monosaccharides. The system with only *pFuc* showed a crossed response with galactose, irrespective of its concentration (Figure 2A). This result is in part explained by the vigorous growth on galactose (Figure 2E). Low concentrations of glucose and mannose (5 and 10 mM), but not higher, also triggered GFP production (Figure 2B,D). Sialic acid appeared not to induce GFP expression (Figure 2C). These results indicate that the sole inclusion of the *pFuc* promoter is insufficient to provide a specific response to fucose. 

**Figure 3:**
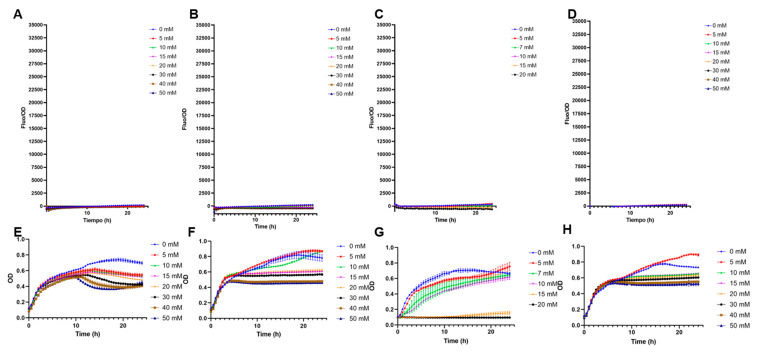
Specificity tests of *E. coli* BL21 containing pFuc_FucR_colE1, using sfGFP as a reporter. (**A**) F/OD values in the presence of increasing concentrations of galactose; (**B**) glucose; (**C**) sialic acid; (**D**) mannose; (**E**) growth curves (OD values) of this strain in the presence of increasing concentrations of galactose; (**F**) glucose; (**G**) sialic acid; (**H**) mannose. Kinetics and growth curves were performed in triplicates and are presented as average ± SD.

Interestingly, the inclusion of the transcription factor increased biosensor specificity (Figure 3). No positive F/OD values were obtained in the presence of galactose, glucose, sialic acid, or mannose (Figure 3). Specificity to these carbohydrates was highlighted by the good growth the biosensor showed in these sugars, with no fluorescence emitted (Figure 3). Finally, rhamnose is another 6-deoxyhexose sugar that could interfere with fucose sensing. A small crossed response was observed for rhamnose, indicating the molecular system needs further improvements in its specificity (Supplementary Figure S2). 

### 3.3. Calibration Curves

**Figure 4:**
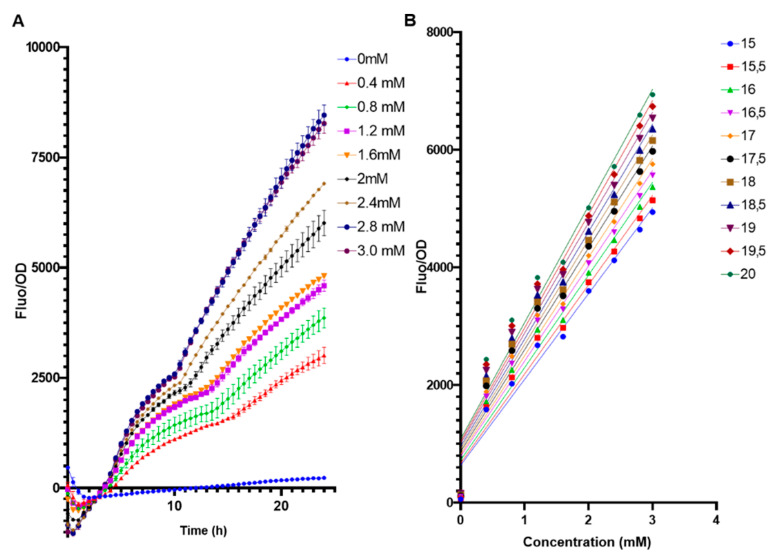
(**A**) Standardized F/OD values of *E. coli* BL21 pFuc + FucR at different concentrations of fucose for 24 h, in the range of 0–3 mM fucose. All values are presented as average ±SD; n = 3. (**B**) Calibration curves of F/OD vs. fucose concentration for the *E. coli* BL21 pFuc+ FucR biosensor from 0 mM to 3 mM for 20 h. Numbers on the right indicate time points (h).

The biosensor *E. coli* BL21 pFuc + FucR high copy (colE1) was evaluated in a range of 0 to 3 mM of fucose to assess its performance under low concentrations (Figure 4). Even at 0.4 mM fucose, the system generated a measurable output (Figure 4A). It can be observed that from 15 h and after, fucose concentrations were well differentiated, with a positive linear correlation between fucose amounts and F/OD values (Figure 4B). Applying a linear regression to these parameters (Supplementary Table S1), the best correlation (higher R squared value) was obtained at 15.5 h (Figure 4B, Supplementary Table S1).

### 3.4. Sensitivity of the E. coli BL21 pFuc+FucR colE1 Biosensor

**Figure 5:**
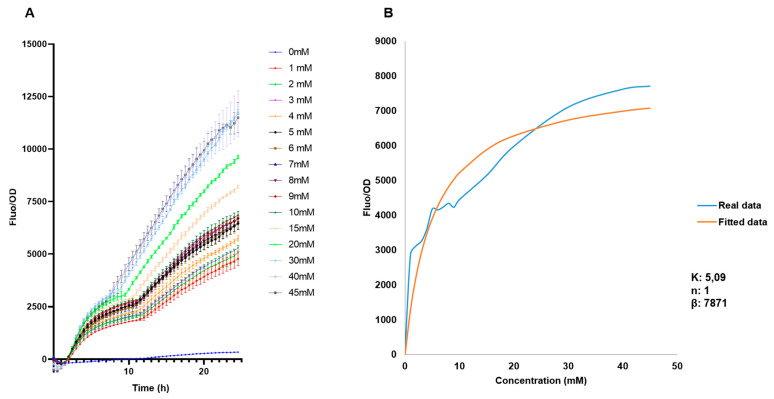
(**A**) Standardized F/OD values of *E. coli* BL21 pFuc + FucR at different concentrations of fucose for 24 h, in the range of 0–45 mM fucose. (**B**) Biosensor response and fitted response to Hill equation parameters for an activator. Concentrations of K are mM, n has no units, and β has units of h^−1^.

The behavior of the biosensor was later evaluated in a broader range to be used for measurements of free fucose, from 0 mM to 45 mM (Figure 5A). As expected, increasing F/OD values were obtained. These data were used to determine the regulatory parameters of the Hill equation ([42]; see methods). F/OD values at 15.5 h were used to fit experimental data to the equation. Modeling results indicate a Hill coefficient value n of 1, which suggests that FucR regulates its promoter via simple non-cooperative regulation. This suggests that FucR binds only one fucose molecule upon binding its DNA. K is a dissociation constant, and a small value was obtained (5 mM). K indicates the affinity of FucR for its promoter, representing the concentration of fucose required to activate 50% of the maximal response. 

### 3.5. Measuring Fucose in the Supernatant of B. bifidum JCM1254

**Figure 6:**
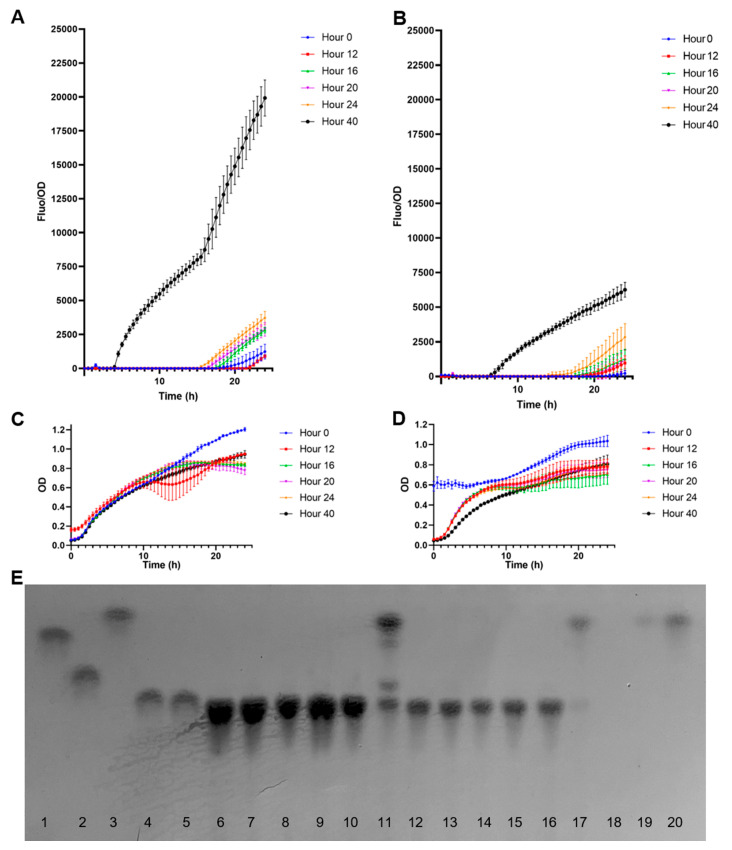
Quantification of fucose in *B. bifidum* supernatants using the biosensor *E. coli* BL21 pFuc + FucR colE1. (**A**) Normalized F/OD values for supernatants after growth in 2FL; (**B**) Normalized F/OD values for supernatants after growth in 3FL; (**C**) growth curves of the biosensors in the presence of supernatants *from B. bifidum* grown in 2FL; (**D**) growth curves of the biosensors in the presence of supernatants *from B. bifidum* grown in 3FL; (**E**) TLC analysis of *B. bifidum* supernatants. Standards used were 1: galactose, 2: lactose, 3: fucose, 4: 2FL, 5: 3FL, at 1 mg/mL. 6–11: supernatants from 2FL growth at 0 (6), 12 (7), 16 (8), 20 (9), 24 (10), and 48 h (11). 12–17: supernatants from 3FL growth at 0 (12), 12 (13), 16 (14), 20 (15), 24 (16), and 48 h (17). Lane 19 corresponds to 1 mM fucose, and lane 20 to 10 mM fucose. Kinetics and growth values shown correspond to average ± SD; n = 3.

Finally, the biosensor was used to measure fucose concentrations from a bacterial supernatant (Figure 6). *B. bifidum* can ferment HMOs, especially 2FL and 3FL, as carbon sources. This microorganism displays extracellular α1-2 and α1-3 fucosidase activities, releasing free fucose in the medium and allowing the bacterium to use lactose [29]. The bacterium was cultured anaerobically for 40 h in a medium supplemented with either 2FL or 3FL. Samples were taken regularly (Figure 6) and incubated with the *E. coli* biosensor. OD and fluorescence measurements were taken for 24 h at an interval of 30 min. 

Figure 6A shows normalized F/OD values of the supernatants obtained from *B. bifidum* growing on 2FL. Supernatants from time points at 12–24 h generated low but increasing F/OD values in time (Figure 6A). A strong fucose signal was detected at 40 h. These results correlated well with a visual assessment of carbohydrates in TLC (Figure 6E), where a strong band with the same migration as fucose was observed. Finally, no major differences in growth were observed for the biosensors using the supernatants from multiple time points, suggesting they were not inhibitory. 

In the case of *B. bifidum* supernatants with 3FL, a similar result was observed compared to 2FL (Figure 6B). The released fucose concentration at 40 h was lower than in 2FL (Figure 6B), and the supernatant sample at 24 h also showed a significant fluorescent output. Similar to 2FL supernatants, only the 40 h sample showed a strong fucose band in the TLC, which correlated with fluorescence data. 

At 15.5 h of incubation, the biosensor incubated with the 2FL supernatant at 40 h presented a normalized F/OD value average of 8206.12, while for 3FL at 40 h was 3717.66 after 15.5 h. These values were used in a calibration curve obtained from the linear regression analysis (Figure 5A). The extrapolation of fucose concentrations in these supernatants was 42.4 mM for 2FL and 6.47 mM for 3FL. These values correlated well with TLC band intensity and appeared in the correct range compared with fucose standards of 1 and 10 mM (Figure 6E).

## 4. Discussion

In this study, we constructed a biosensor for quantifying fucose in biological samples, using a molecular promoter and transcription factor naturally occurring in *E. coli* and using sfGFP as output. *E. coli* is well characterized by its L-fucose utilization mechanism [36]. A permease allows fucose entrance, and a feeder pathway allows L-fucose conversion into lactate and 1,2-propanediol, generating NADH and FADH [35]. An intermediate in this pathway, fuculose-1-phosphate, is the ligand recognized by the system regulator, FucR [37]. Therefore, our biosensor is expected to sense fuculose-1-phosphate and not directly fucose. The system requires that any external fucose sensed be first metabolized to generate an output. 

Fucose is not among the most preferred carbon sources for *E. coli*, compared to glucose, galactose, or arabinose [43,44]. It shows a slow growth in this substrate in minimal media [35]. Catabolic repression exerted by CRP on the fucose promoter is also complemented with small RNA regulation via Spot42 [45]. The biosensor developed here is based on the activation role of FucR, which binds its promoter in the presence of fuculose-1-phosphate and allows the expression of sfGFP. We were able to determine in this study that FucR displays simple regulation, showing no cooperativity and suggesting it acts as a monomer. It is known that rhamnose appears to induce the operon [46], and fucose can also activate the galactose *galETK* system in *E. coli* [43]. These findings indicate that crossed regulatory responses of fucose and FucR are common in *E. coli* and might alter biosensor specificity. 

Results in this study showed a high increase in specificity attributed to the presence of FucR. *pFuc* alone showed little specificity, indicating that other molecules can still induce leaky expression. The cloning of additional copies of FucR dramatically reduced crossed regulatory responses, probably increasing the threshold of fucose activation and resulting in a much tighter response. Finally, a much better resolution for strain BL21 compared to DH5α could be explained by the mutation in the *lon* protease in BL21, which allows a smaller reporter protein degradation and increased half-life [47]. 

The biosensor characterized showed a good linear response in the low concentration range (0–3 mM) or higher (0–45 mM). Some applications of the biosensor are as a diagnostic tool. Fucose is metabolized in the liver, and excess fucose is secreted in the urine [2,48]. A rare genetic disorder is fucosidosis, where fucose found in glycoconjugates cannot be removed and accumulates in the body resulting in severe consequences [49]. Therefore, quantifying fucose in urine could be of interest, especially since there is an increase in fucose concentrations in certain liver diseases [19,50]. 

Another field of application of biosensors is in GIT and gut microbiome research [21]. The rapid quantification of HMOs in breast milk samples is desirable, especially fucosylated molecules. Similarly, there is great interest in the enzymatic biosynthesis of these molecules, which requires quantifying fucose [51]. Finally, several gut microbes display α-fucosidase activities and use fucose as a carbon and energy source [6,9,52]. *Bifidobacterium* and *Bacteroides* species are well known for their extracellular activities, which release fucose from HMO, mucins, other glycoproteins, or glycolipids [3,10]. Therefore, free fucose can be expected to be detected in GIT contents in mammals. Free fucose is also known to participate in cross-feeding interactions, where the fucose released by one microorganism is imported and used by another. This has been observed during the consumption of mucin glycans and HMO, for example, between *B. bifidum* and *Bifidobacterium breve* [12,13,53]. Therefore, an accurate and inexpensive method for quantifying fucose could show how this monosaccharide is shared between species. In this study, the developed biosensor displayed a good performance in quantifying fucose derived from 2FL utilization by *B. bifidum* and could be used in studying cross-feeding interactions. 

## 5. Conclusions

A fluorescent quantification method of fucose was developed in this study in *E. coli* with a high copy plasmid containing a reporter sfGFP, a fucose promoter, and FucR. The biosensor showed good sensitivity and specificity, showing a linear response to increasing fucose concentrations from 0 to 45 mM, a range within physiological concentrations. A validation to quantify fucose in a bacterial supernatant during HMO utilization was achieved. This method could be coupled to other enzymes (fucosidases, endoglycosidases, peptidases) to determine the concentration of fucosylated glycoconjugates. This assay could be an important tool for clinical research (serum, urine), foods, bioprocessing in the production of fucosylated glycoproteins, or detecting fucose in bacterial supernatants and quantifying fucose in fecal samples or bioreactors.

## Figures and Tables

**Table 1:** Plasmids used in this study. These plasmids were used to transform bacteria that did not metabolize fucose as a carbon source. BL21 and DH5α colonies were obtained for these plasmids.

	Plasmids	Antibiotic	Description
1	Pfuc *sfGFP_*ColE1	Ampicillin	High-copy plasmid colE1 with fucose promoter and sfGFP with an ampicillin resistance gene.
2	Pfuc *sfGFP_*SC101	Chloramphenicol	Low-copy plasmid SC101 with fucose promoter and sfGFP with a chloramphenicol resistance gene.
3	Pfuc+FucR1_colE1	Ampicillin	High-copy plasmid colE1 with transcription factor FucR, fucose promoter and sfGFP with an ampicillin resistance gene

## Data Availability

The data presented in this study are available upon request.

## Supplementary Materials

The following supporting information can be downloaded at: https://www.mdpi.com/article/10.3390/bios13030388/s1, Table S1. Linear regression of the calibration curves obtained for 0 mM to 3 mM with a resolution of 0.4 mM at different incubation times; Figure S1. Representation of the plasmids used in this study. A: pFUC_sfGFP_colE1 is a high copy plasmid containing the fucose promoter controlling GFP expression and an ampicillin resistance gene; B: pFUC_sfGFP_SC101 is a low copy plasmid containing the fucose promoter controlling GFP expression and a chloramphenicol resistance gene: C: pFUC+FucR1_colE1 is a high copy plasmid containing the fucose promoter controlling GFP expression, in addition to a cloned FucR encoding gene and an ampicillin resistance gene. Internal arrows correspond to transcription units; Figure S2. Specificity tests of E. coli BL21 containing pFuc_FucR_colE1, using sfGFP as a reporter, for rhamnose. A: F/OD values in the presence of increasing concentrations of rhamnose; B: growth curves (OD values) of this strain in the presence of increasing concentrations of rhamnose. Kinetics and growth curves were performed in triplicates and are presented as average ± SD.
