# Supplementary material for: Developing a Fluorescent Inducible System for Free Fucose Quantification in Escherichia coli
Source: Biosensors (Basel). 2023 Mar 15;13(3):388. doi: 10.3390/bios13030388 (PMC10046853; doi:10.3390/bios13030388)
Supplement: Supplementary file 1 [file biosensors-13-00388-s001.zip › biosensors-2247472-supplementary.pdf]

Supplementary Material

Developing a Molecular Biosensor of Free Fucose in Escherichia coli.

Samantha Nuñez, María Barra and Daniel Garrido *

Department of Chemical and Bioprocess Engineering, School of Engineering, Pontificia Universidad Católica de Chile, Vicuña Mackenna, 4860, Santiago, Chile.

* Correspondence: dgarridoc@ing.puc.cl.

Table S1. Linear regression of the calibration curves obtained for 0 mM to 3 mM with a resolution of 0.4 mM at different incubation times.

Hour	Equation	R-squared
15	Y = 1459X + 640,8	0,9682
15,5	Y = 1517X + 674,9	**0,9684**
16	Y = 1574X + 727,1	0,967
16,5	Y = 1619X + 792,6	0,9648
17	Y = 1675X + 827,4	0,9643
17,5	Y = 1732X + 872,5	0,9636
18	Y = 1779X + 915,2	0,9624
18,5	Y = 1826X + 959,9	0,9616
19	Y = 1877X + 1010	0,9606
19,5	Y = 1931X + 1045	0,9596
20	Y = 1981X + 1086	0,9588

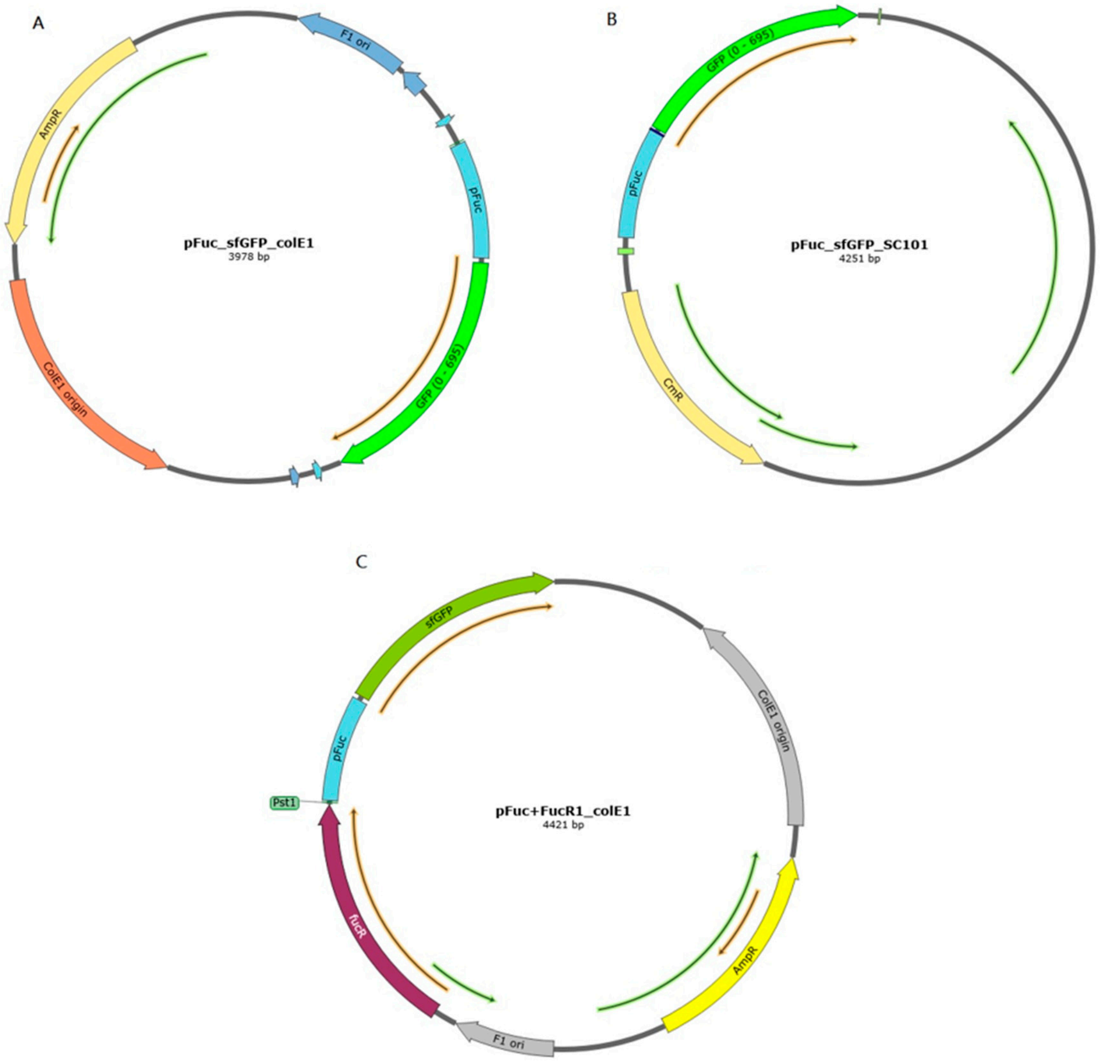

Figure S1. Representation of the plasmids used in this study. A: pFUC_sfGFP_colE1 is a high copy plasmid containing the fucose promoter controlling GFP expression and an ampicillin resistance gene; B: pFUC_sfGFP_SC101 is a low copy plasmid containing the fucose promoter controlling GFP expression and a chloramphenicol resistance gene: C: pFUC+FucR1_colE1 is a high copy plasmid containing the fucose promoter controlling GFP expression, in addition to a cloned FucR encoding gene and an ampicillin resistance gene. Internal arrows correspond to transcription units. .

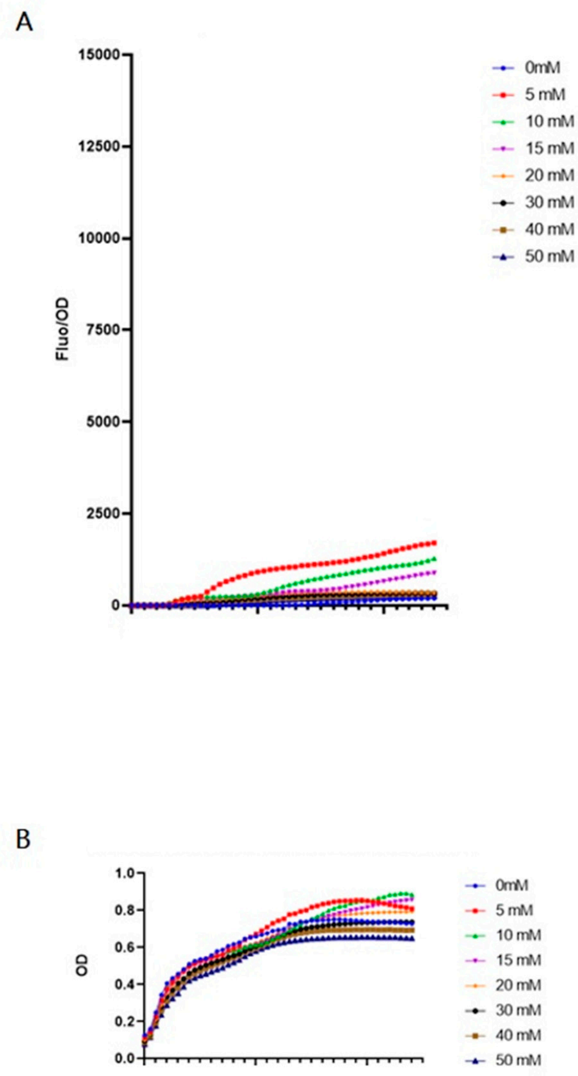

Figure S2. Specificity tests of *E. coli* BL21 containing pFuc_FucR_colE1, using sfGFP as a reporter, for rhamnose. A: F/OD values in the presence of increasing concentrations of rhamnose; B: growth curves (OD values) of this strain in the presence of increasing concentrations of rhamnose. Kinetics and growth curves were performed in triplicates and are presented as average ± SD.
